# Applications of Nanofabrication Technologies in the Preparation of Biomimetic Structures

**DOI:** 10.3390/biomimetics11080562

**Published:** 2026-08-06

**Authors:** Hongwen Sun, Baohua Yang, Xiaomin Xie, Lei Li, Hengmei Li, Jie Shen

**Affiliations:** School of Artificial Intelligence, Changzhou Vocational Institute of Mechatronic Technology, 26 Middle Mingxin Rd., Changzhou 213164, China

**Keywords:** nanofabrication, biomimetic structures, bio-templating, replication, surfaces, bionic devices, cross-scale, biosensors

## Abstract

Biomimetic structures are now a major topic of research, as natural systems achieve high performance through hierarchical organization, multifunctional interfaces, and scale-bridging design principles. Nanofabrication provides a powerful approach to recapitulate biological architectures from the nanoscale to the macroscale, allowing accurate control of the surface chemistry, geometry, transport, mechanics and function. Recent work demonstrates that this approach is especially critical for bionic devices and systems, including biosensors, drug delivery platforms, tissue-engineered constructs, organ-on-chip systems, soft robots, and biohybrid devices. The aim of this review is to provide a systematic overview on how nanofabrication allows the construction of biomimetic structures, with emphasis on bio-templating and replication of natural structures, applications of nanofabrication in bionic devices and systems, and cross-scale biomimetics.

## 1. Introduction

Biomimetics is an interdisciplinary field that is inspired by the structures, properties and functions that living organisms have developed over millions of years. Nature provides a vast library of astonishing architectures with extraordinary mechanical, optical and chemical functionalities, such as superhydrophobic lotus leaf, adhesive gecko foot, and iridescent Morpho butterfly wing [1,2,3]. Biomimetic structures are designed to mimic or adapt these natural designs for the purpose of engineering materials and devices with improved or novel performance.

Since prehistoric times, humans have looked to nature’s designs, with early civilizations observing and copying the natural world to solve practical problems. The bow and arrow, for example, drew on the slingshot-like motion of plant tendrils, while aqueducts echoed the efficient water transport seen in plant vasculature. This kind of intuitive bioinspiration—spotting a natural phenomenon and building a simplified human version—has run through history and led to some of our most transformative technologies. The Renaissance marked a clear step forward in bioinspired design, as polymaths like Leonardo da Vinci began to systematically study animal anatomy to guide their engineering work. His sketches of flying machines, grounded in the morphology of bird wings, and his submarine concepts, inspired by fish movement, point to an early grasp that evolution had been optimizing forms and functions over millions of years. Still, these initial efforts were constrained by the materials and manufacturing methods of the time, yielding prototypes that could only approximate, rather than truly replicate, the performance of their biological models.

With the Industrial Revolution came a different approach: mechanical systems started to outdo natural organisms in specific tasks. Steam engines could generate more power than biological muscles, and airplanes could fly faster than birds. In this climate, interest in bioinspired research faded somewhat, as engineers turned their attention to refining mechanical systems instead of mimicking biological ones. Even so, a few fields kept the link to natural designs alive—naval architecture being a notable case, where ship hulls continued to borrow from fish body shapes for hydrodynamic efficiency.

The late 20th century saw a renewed wave of interest in bioinspired design, fueled largely by two developments: the maturation of interdisciplinary science and the arrival of new manufacturing technologies. Scientifically, deeper insights from biology, materials science, and engineering began to clarify just how biological systems achieve their striking performance. Take the gecko’s foot: discovering that its adhesion rests on van der Waals forces, not suction or chemical bonds, explained how these animals scale vertical surfaces. That finding directly inspired the creation of dry adhesives with reversible attachment, now applied in robotics and aerospace. At the same time, nanotechnology and advanced fabrication gave researchers the tools to turn biological principles into engineered systems. Where traditional methods like casting and machining had been limited to macroscale features, newer nanofabrication techniques—lithography, self-assembly, additive manufacturing—made it possible to build structures with feature sizes matching those in nature, from the hierarchical architecture of bone to the antireflective surfaces of moth eyes. This merging of scientific understanding and technological capability launched what could be called a “biomimetic renaissance”—a period of fast-paced progress in bioinspired design across many fields. Both government agencies and private companies saw the innovative potential here, leading to dedicated research centers and substantial funding. The European Union’s Framework Programmes, for example, have backed a range of bioinspired projects, while companies such as Boeing and Airbus have put resources into biomimetic research aimed at improving aircraft efficiency and cutting noise.

Bringing biomimetics together with nanofabrication has opened fresh pathways for constructing structures that closely approach the complexity and performance of their natural counterparts. Nanofabrication encompasses a set of advanced techniques for manipulating materials and devices at the nanometer scale, typically below 100 nm, and stands as a cornerstone of modern nanotechnology and nanoscience [4,5,6,7,8,9,10]. The field has evolved quickly, now enabling the creation of intricate, functional structures with remarkable precision and repeatability. These technologies have reshaped fields from electronics and photonics to energy and, perhaps most notably, biomedicine, by offering routes to design and mass-produce complex systems with tailored shapes and functions [11,12,13,14,15,16]. Because nanofabrication allows precise control over material shape, size, and arrangement at the nanoscale, it is particularly suited to replicating the hierarchical, multifunctional architectures found in nature. This synergy has produced advanced materials and devices for tissue engineering, drug delivery, biosensing, and beyond. The cross-technology synergy of nanofabrication and biomimetic design has led to materials and devices that not only mimic the hierarchical organization and multifunctionality of natural systems but also exceed them in tunability, reproducibility, and scalability [17].

**Figure 1:**
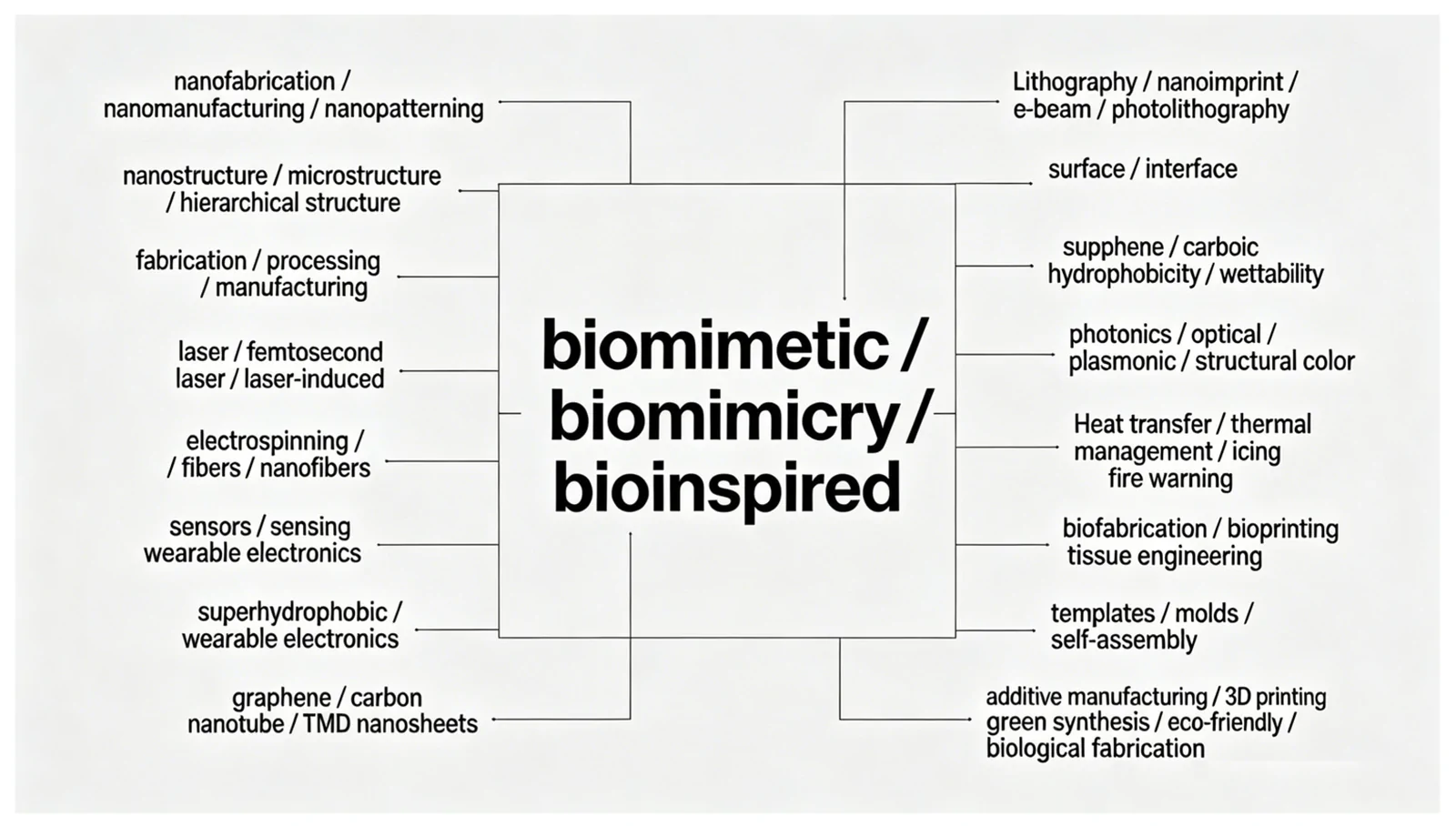
Knowledge graph of biomimetic nanofabrication technologies.

As Figure 1 illustrates, research into nanofabrication and bioinspired structures is inherently multidisciplinary, drawing on biology, physics, chemistry, materials science, and engineering. Biologists unpack the form–function relationships in natural systems and pinpoint principles that can be translated into designs. Physicists and chemists work out the fundamental processes and materials that underlie nanofabrication techniques, while materials scientists tune these materials for specific uses. Engineers, in turn, integrate the components into working systems that tackle real-world problems in energy, medicine, robotics, and related areas. This interdisciplinary character is both a strength and a challenge. It spurs innovation by encouraging ideas to cross-pollinate across fields, but it also demands that researchers develop fluency in multiple domains—a time-consuming and complex task. Given this, a comprehensive review that maps the relationship between nanofabrication and bioinspired structures is needed, both to save time and to help researchers quickly grasp the relevant information.

Professor Ivanova et al. systematically reviewed the current understanding of mechanobactericidal mechanisms, by integrating both the key contributing factors and the diverse experimental techniques that underpin the proposition of these mechanisms. They further presented a critical analysis of the inconsistencies in the evaluation frameworks for nanostructured surfaces across the existing literature [18]. Zhang et al. systematically elaborates on the design strategies and functional characteristics of diverse biomimetic antibacterial materials, with a focused highlight on their translational therapeutic potential in anti-infection clinical scenarios [19]. An injectable nanofiber composite self-healing hydrogel was developed by Tian et al. for treating infected wounds. Experimental results demonstrated that the hydrogel exhibited exceptional biocompatibility and potent antimicrobial activity [20]. Liu et al. focused on nanoimprint lithography (NIL) and nanotransfer printing (nTP), showing how these micro- and nanofabrication tools can boost biosensing performance through improved nanostructures that enable high-sensitivity detection and multifunctional use [21]. Dubey et al. took a deep look at various self-assembly routes for fabricating nanostructures, with an emphasis on DNA origami, block copolymers, and colloidal assembly, and touched on their applications in drug delivery, biosensing, nanofabrication, and computational data storage [22]. The Manimaran team examined advances in metal nanofabrication that rely on microbial exopolysaccharides, opening promising directions for nanotechnology-based strategies against cancer and microbial infections [23]. Yang and Ma’s group surveyed multiphoton polymerization (MPP)-based micro/nanomanufacturing, stressing its high precision and noncontact advantages for precision medicine, and organized the biomedical applications into delivery, tissue modeling, surgery, and diagnosis while addressing hurdles such as material design and process optimization [24]. Their work points toward practical pathways for moving MPP technology into clinical settings. A review by the Zhang team explored recent progress in bionic optical materials (BOMs), highlighting their bioinspired design principles and strong broadband extinction performance, and assessed their uses in optical sensing and imaging while flagging emerging directions like nanofabrication and machine learning-driven optimization [25]. Lee et al. analyzed biomimetic surfaces as a nature-inspired route to improve medical and implantable devices, covering recent micro- and nanoscale manufacturing advances that allow precise surface modifications to mimic biological antifouling, antibacterial, and adhesive properties [26]. Their review brings out the potential of biomimetic coatings to enhance biocompatibility, reduce inflammation, and support stable tissue integration in long-term clinical applications. Although above recent reviews have covered the convergence of nanofabrication and biomimetics—often with a focus on biosensors, drug delivery, or specific surface functionalities—the field still lacks a review that systematically addresses how nanofabrication enables biomimetic systems across length scales, particularly in the rapidly expanding application territories of organ-on-chip platforms, biohybrid tissues, wearable electronics, and soft robotics. In biological systems, performance seldom arises from a single feature; it emerges from the way nanoscale chemistry, microscale organization, and macroscale geometry coordinate. Capturing that coordination in engineered structures demands a fabrication logic that goes beyond single-scale patterning. The present review builds its argument around this cross-scale biomimetics framework. By pulling together advances that have been scattered across disparate device communities and highlighting the translation from nanoscale features to integrated working systems, we aim to fill a gap that existing reviews have left open.

The remainder of this review is organized as follows. Section 2 provides a concise, critically oriented overview of the nanofabrication techniques most relevant to biomimetics, emphasizing their capabilities and limits. Section 3 focuses strictly on the design principles of biomimetic materials, tracing the shift from shape imitation to multi-scale function integration. Section 4 narrows the lens to surfaces and interfaces, where hierarchy and patterning produce properties such as superhydrophobicity and antifouling. Section 5 discusses bio-templating as a distinct replication strategy that bridges natural templates and synthetic constructs. Section 6 expands the perspective to system-level bionic devices and platforms, while Section 7 synthesizes the preceding themes under the unifying concept of cross-scale biomimetics. Finally, Section 8 discusses the challenges and future directions of this field, identifying key bottlenecks and emerging opportunities.

To build the core body of literature for this review, we searched ScienceDirect using combinations of terms that reflect the scope of the manuscript. The primary keywords included nanofabrication, biomimetic structures, bio-templating, replication, surfaces, bionic devices, cross-scale, biosensors, and lithography, connected through Boolean operators (AND, OR) to capture the intersection between fabrication methods and biomimetic applications. Typical search strings took the form (nanofabrication OR lithography OR nanoimprint OR electrospinning) AND (biomimetic OR bio-inspired OR bionic) AND (surfaces OR structures OR devices). The search was set to cover the period from January 2016 to June 2026, as the intention was to ground the discussion primarily in the most recent developments while still including a limited number of earlier, foundational studies identified through citation tracking. We screened titles and abstracts for direct relevance to nanofabrication-enabled biomimetic structures; articles that dealt only with biological characterization, lacked a clear fabrication component, or were purely computational without experimental validation were excluded. The initial electronic search was then supplemented by backward and forward citation searches from key papers, which helped recover important early works and also capture emerging directions that appeared after our primary search window. The final collection of references reflects this two-stage process and is intended to be representative of the field rather than exhaustive.

## 2. Nanofabrication Techniques

Nanofabrication encompasses a suite of patterning and structuring methods that create features with at least one dimension below 100 nm, but in biomimetic contexts the boundary with microfabrication and additive manufacturing often blurs because biological structures span multiple scales [27,28,29,30]. We use nanofabrication for techniques that reliably generate sub-micrometer features (photolithography, electron-beam lithography, nanoimprint lithography, etc.), whereas microfabrication covers processes whose resolution is typically above 1 µm. Additive manufacturing (e.g., 3D printing) and biofabrication (e.g., cell-laden bioprinting) operate at larger scales but can be hybridized with nanofabrication to build hierarchical scaffolds and devices. Electrospinning and soft lithography sit at the interface: they can produce nanoscale fibers or patterns, yet are often deployed to construct meso- to macroscale architectures. This section therefore groups techniques by their physical principle rather than by a rigid size cut-off, always noting the scale at which each method exerts its primary control. A variety of nanofabrication techniques exist, and many can be adapted or developed specifically to produce biomimetic structures.

Nanofabrication strategies are generally divided into top-down and bottom-up approaches. Top-down methods, such as lithography and etching, trace their roots to semiconductor manufacturing and work by patterning and removing material to arrive at the desired nanostructure [31]. The process typically involves two main stages: forming an image or pattern on a material surface (using a resist sensitive to light, electrons, or X-rays), followed by surface processing and patterning through etching or deposition. These capabilities underpin nanoelectromechanical systems (NEMS), nanophotonic devices, and a broad spectrum of biomedical applications. Bottom-up approaches, including molecular self-assembly and DNA origami, depend on the spontaneous organization of molecules into ordered structures, often driven by specific chemical interactions [32].

Hybrid top-down/bottom-up approaches merge conventional lithography with the self-assembly of biological nanoscale building blocks—virus-derived nanoparticles, for instance—to construct hierarchical structures that span both micro- and nanoscale dimensions. Such hybrid strategies are finding growing use in the fabrication of biomimetic structures that bring together features across multiple length scales.

### 2.1. Lithography-Based Methods

Photolithography transfers a geometric pattern from a photomask to a photosensitive resist by UV exposure [33,34,35]. It is the workhorse of micro- and nanofabrication, delivering reliable nanometer resolution over entire wafers with high throughput. Photolithography is ideal for creating regular arrays of pillars, wells, and channels that serve as cell-culture platforms, biosensor transducers, and microfluidic masters. Its precise alignment capability permits multi-layer structuring that mimics the compartmentalisation seen in tissues. The process is inherently 2.5D; true 3D curved topographies (e.g., bone trabeculae, compound eye curvature) are impossible to produce without grayscale masks or reflow steps, and even then the achievable shapes are limited. The high capital and running costs also restrict rapid prototyping. Consequently, photolithography alone cannot reproduce hierarchical, highly porous biological architectures such as cancellous bone or leaf vein networks.

Electron-beam lithography (EBL) writes patterns directly into an electron-sensitive resist with a focused electron beam, achieving sub-10 nm resolution without a mask [36,37,38,39]. EBL is unrivalled for prototyping master templates of biomimetic nanostructures—such as anti-reflective moth-eye arrays, plasmonic biosensors, and nano-patterned substrates for studying cell-material interactions. The maskless workflow permits rapid design iteration. However, it is a serial process with extremely low throughput and high cost, making it impractical for scaling up biomimetic surfaces to clinically or industrially relevant areas. Proximity effects and substrate charging further complicate patterning on insulating materials common in biomedical devices.

Focused ion beam (FIB) uses accelerated ions (typically Ga^+^ or plasma ions) to mill, deposit, or image materials [40,41,42]. FIB is indispensable for site-specific cross-sectioning of bio-inspired composites, preparing TEM lamellae from biological-synthetic interfaces, and prototyping single nanopores or nanofluidic channels that mimic biological ion channels. The technique is serial and destructive; ion implantation and amorphisation can alter material properties. Throughput is far too low for routine fabrication of bionic devices, and gallium contamination may compromise biocompatibility unless carefully managed.

Nanoimprint lithography (NIL) mechanically presses a hard mould into a polymer resist, replicating features as small as ~10 nm with moderate cost and high throughput [43,44,45]. Both thermal and UV-curing variants exist. The technique can faithfully duplicate natural surface textures—e.g., shark-skin riblets, lotus-leaf papillae, or insect wing nanostructures—by casting them directly or using bio-templated moulds. It also works on flexible substrates, making it attractive for wearable bionic sensors. For this technique, mould wear, demoulding defects, and residual layer thickness control are persistent challenges. Large-area conformal patterning on non-planar, soft, or hydrated biological substrates remains problematic. The technique is poorly suited to generating the through-thickness porosity required for many tissue-engineering scaffolds.

Soft lithography employs elastomeric stamps (usually poly(dimethylsiloxane), PDMS) to transfer patterns, mould structures, or guide fluid flow [46,47,48,49]. It routinely achieves 30 nm resolution, is inexpensive, and can pattern non-planar surfaces and biomolecules. The method excels at fabricating microfluidic networks that recapitulate vascular geometries, organ-on-chip compartments, and topographically patterned substrates for cell alignment. Its ability to print functional inks and biomolecules has enabled spatially controlled co-culture and gradient mimics. However, PDMS stamps can deform, swell, or collapse, limiting pattern fidelity and repeatability over large areas. Resolution is ultimately constrained by the mechanical properties of the elastomer, and producing truly multiscale structures requires combination with other techniques. Generating dense, high-aspect-ratio nanostructures (e.g., gecko setae) with soft lithography alone is challenging.

### 2.2. Film Deposition and Etching

Film deposition technologies cover a family of methods for forming thin films on substrates, broadly divided into physical and chemical routes [50,51,52,53]. Physical vapor deposition (PVD) and chemical vapor deposition (CVD) are the workhorses, while advanced variants such as atomic layer deposition (ALD) and pulsed laser deposition (PLD) deliver tighter control over film thickness, composition, and uniformity. Recent efforts have targeted improvements in scalability, film quality, and conformality, supporting uses in semiconductors, optoelectronics, and energy devices. Techniques like ALD and plasma-enhanced processes now enable atomic-level control and high-quality coatings on complex topographies, meeting the demands of modern micro- and nanofabrication.

Etching plays a critical role in pattern transfer and material removal during micro- and nanofabrication [54,55,56]. Reactive ion etching (RIE) employs energetic, chemically reactive ions to strike a balance between physical sputtering and chemical etching, giving control over selectivity and isotropy for materials that include SiC and silicon-based substrates. Inductively coupled plasma (ICP) etching, an advanced variant of RIE, decouples ion energy from ion density, yielding higher plasma densities, faster etch rates, improved anisotropy, and better selectivity—qualities that make it well suited to deep and complex patterning.

Conformal ALD coatings can functionalise porous scaffolds without blocking pores, and ICP etching can carve high-aspect-ratio nanostructures such as silicon nano-pillars for bactericidal surfaces. However, deposition and etching alone cannot build a biomimetic architecture; they must be coupled with lithography or templating. Process compatibility with thermally sensitive or hydrated biomaterials is also an issue.

### 2.3. Laser-Based Methods

Pulsed laser ablation removes material to create surface textures, while two-photon polymerization (2PP) and related direct-laser-writing techniques additively build complex 3D nanostructures with features down to ~100 nm [57,58,59,60].

Laser ablation can engrave superhydrophobic or drag-reducing surface morphologies inspired by lotus leaves and shark skin onto metals, ceramics, and polymers. 2PP is one of the few techniques that can produce truly 3D micro- and nano-lattices, trabecular networks, and compound-eye-inspired lens arrays in a single step [61,62,63,64]. More recently, ultrafast lasers have shown promise in shrinking heat-affected zones and producing high-precision, low-roughness microstructures [65,66].

Ablation can leave heat-affected zones, altering surface chemistry. 2PP suffers from slow build speed and a limited palette of photoresists with suitable mechanical and biological properties. Scaling 2PP to macroscopic dimensions while preserving nanoscale features remains a major hurdle.

### 2.4. 3D Printing and Additive Manufacturing

Extrusion-, inkjet-, and light-based 3D printers deposit material layer-by-layer with resolution ranging from a few micrometres (micro-stereolithography) to hundreds of micrometres (fused deposition modelling) [67,68,69,70]. Although most are not “nano” by strict definition, they are frequently integrated with nanofabrication to produce hierarchical scaffolds.

3D printing excels at producing macroscopic shapes with patient-specific geometries and controlled macro-porosity, such as soft actuators that mimic muscular hydrostats. Additive manufacturing, including 3D printing, makes it possible to fabricate complex biomimetic structures-hierarchical porous scaffolds for bone regeneration and superhydrophilic or superhydrophobic surfaces for water management and bioengineering are two prominent examples [71,72].

Stand-alone 3D printers cannot define the nanoscale surface roughness, chemistry, or nanofibrous texture that cells sense; post-processing with coatings or electrospinning is required. Resolution–throughput trade-offs restrict the simultaneous achievement of nanoscale detail and large build volumes.

### 2.5. Electrospinning

Electrospinning stretches a polymer jet under a high electric field to produce continuous fibres with diameters from ~10 nm to several micrometres [73,74,75,76].

The resulting non-woven mats closely resemble the fibrous architecture of the extracellular matrix, making them popular for tissue-engineering scaffolds, wound dressings, and filter membranes that emulate biological sieving. In biomedicine, electrospun nanofiber scaffolds combine biocompatibility with biodegradability, making them strong candidates for tissue engineering and regenerative medicine. Drug-loaded nanofiber systems further enable targeted delivery, boosting therapeutic efficacy while limiting systemic side effects [77,78,79,80].

Fibre deposition is often random and difficult to control in three dimensions; obtaining aligned or spatially organised fibre arrays (e.g., mimicking tendon or corneal stroma) requires modified collectors or post-processing. The technique alone cannot generate the dense, oriented, and crystallographically registered nanostructures seen in bone or enamel.

### 2.6. Molecular Self-Assembly and DNA Origami

Molecules spontaneously organise into ordered nanostructures via non-covalent interactions, giving near-atomic resolution [81,82,83,84]. DNA origami adds programmability, enabling arbitrary 2D and 3D shapes.

Self-assembly can create biomimetic lipid bilayers, enzyme-mimetic catalytic pores, and photonic nanostructures. DNA origami has been used to construct molecularly precise drug carriers and scaffolds for proteins, mimicking viral capsids and enzyme clusters.

For these bottom-up techniques, long-range order is difficult to sustain while defects accumulate. Translating a designed nanoscale assembly into a macroscopically useful material or device remains a fundamental challenge. Stability under physiological conditions and cost of DNA synthesis also limit current applications.

### 2.7. Complementary Techniques Worthy of Note

Several other techniques are increasingly relevant to biomimetic nanostructure fabrication. Scanning probe lithography (e.g., dip-pen nanolithography) can write chemical patterns with sub-50 nm resolution and is especially useful for depositing biomolecules in registry [85]. Colloidal lithography uses self-assembled monolayers of microspheres as an etch mask to produce periodic nanopillar or nanohole arrays, often for anti-reflective or plasmonic coatings [86]. Block-copolymer lithography exploits microphase separation to generate highly regular 10–50 nm patterns over large areas [87]. Capillary-force lithography fills moulds by capillarity, enabling replication of high-aspect-ratio biological templates on soft substrates [88].

### 2.8. Commercial Viability, Cost, and Scalability Considerations

Moving from laboratory demonstrations to commercial products depends critically on cost, throughput, and process reliability—factors that are rarely reported in the primary literature but determine which techniques can realistically scale. Among the methods surveyed, photolithography remains the only fully mature, high-throughput platform, yet its capital costs confine it to high-value-added products. Soft lithography and electrospinning are inexpensive to establish but suffer from limited stamp lifetime and poor fibre alignment, respectively, restricting their precision manufacturing potential. Nanoimprint lithography is approaching commercial viability for optical films and certain biosensors, whereas electron-beam lithography and focused ion beam milling remain too slow and costly for any application beyond prototyping or mask fabrication. Additive techniques such as two-photon polymerization, and bottom-up methods like DNA origami, offer high resolution but are presently far from cost-effective mass production. A recurring bottleneck is that hybrid cross-scale processes compound capital costs, process complexity, and yield losses with each additional step. A realistic path to industrial adoption for more complex cross-scale systems will require either drastic process simplification or integrated continuous-flow platforms that do not yet exist at scale.

### 2.9. Comparative Table of Various Techniques

In summary, Table 1 presents a comparative overview that condenses the key performance indicators of the aforementioned techniques. This table aims to highlight the unique strengths of each method for reproducing biological structures and clarify their respective limitations.

## 3. Biomimetic Material

This section deals exclusively with the design principles of biomimetic materials, focusing on how biological structure–function relationships are translated into synthetic material systems; processing routes and device applications are treated separately in later sections. Biomimetic materials are increasingly defined less by what they copy and more by how they translate the logic of biological design into structures that can actually be manufactured. Early efforts centered on reproducing single structural motifs—nacre-like brick-and-mortar layering, hierarchical porosity, or surface roughness—mainly to capture high strength, toughness, hydrophobicity, or adhesion [89,90,91]. More recent work points to a clear shift toward multi-scale, function-integrated biomimicry, where the aim is to reproduce coupled properties across nano-, micro-, and macroscales rather than to copy one morphology on its own [92,93,94]. The shift is significant because biological materials derive their performance not from a single component or uniform phase, but from interfaces, gradients, anisotropy, and hierarchical order. In this sense, biomimetic material design has progressed from “shape imitation” to structure–function translation, a perspective that now sits at the center of nanofabrication-enabled biomimetic structures [95].

**Figure 2:**
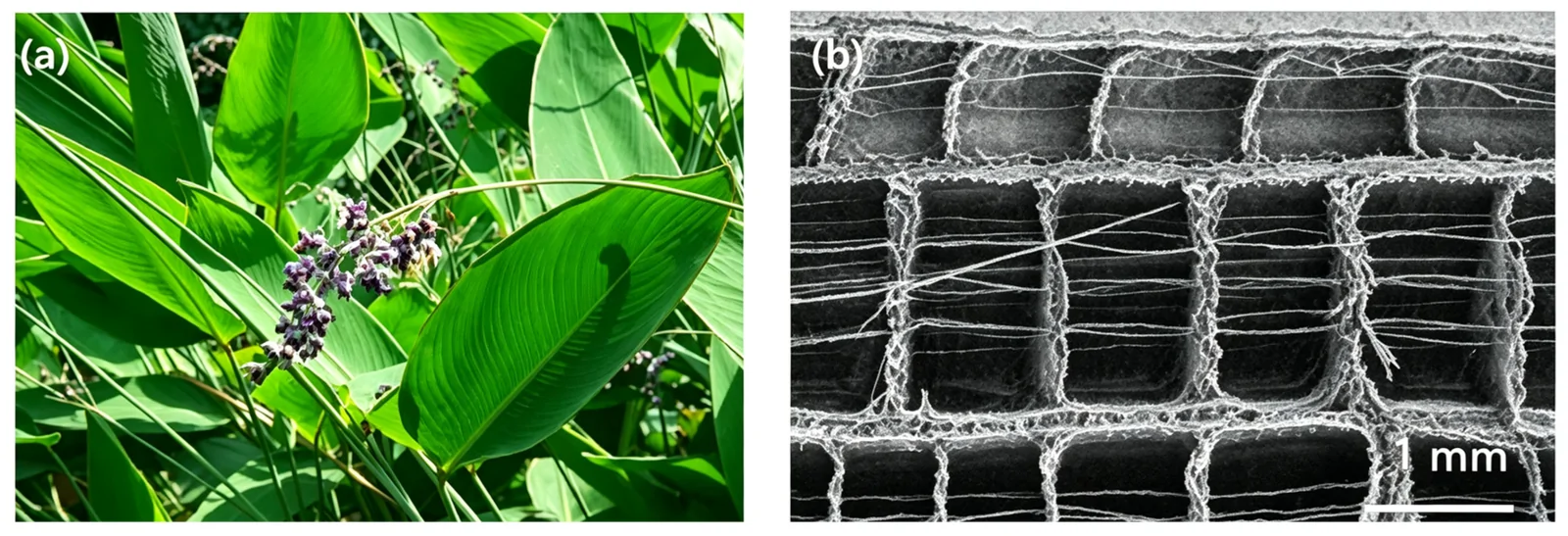
Images of *Thalia dealbata*. (**a**) The leaves; (**b**) SEM image showing the hierarchical architecture.

A dominant theme over the past decade has been the use of natural structures as templates or design references for synthetic materials. Nacre-inspired, bone-inspired, plant-inspired, and sponge-inspired systems have repeatedly demonstrated that ordered heterostructures can outperform conventional homogeneous materials when the architecture is faithfully reproduced [96,97,98,99]. Bidirectional freezing and ice templating, for instance, have been employed to produce aligned lamellar scaffolds and hierarchical porous networks that boost strength, toughness, and resilience while keeping density low [98,99,100,101]. Figure 2 shows the hierarchical architecture of a Thalia dealbata leaf. Along similar lines, cross-scale templating of cellulose nanocrystals and other nanofibers illustrates that nanoscale alignment alone is often not enough; macro-level patterning is required to unlock tougher fracture behavior and more faithful biomimicry [102,103]. A useful contrast emerges: single-scale mimicry tends to enhance one property, whereas cross-scale biomimicry can reconcile trade-offs—stiffness versus toughness, or permeability versus mechanical integrity—that single-scale approaches cannot easily resolve [93,94,104].

Work published between 2023 and 2026 pushes this line of thinking further, placing greater weight on quantitative and programmable biomimetics. Researchers in the field are turning more and more to modeling, optimization, and controlled assembly to turn biological inspiration into reproducible fabrication rules [104,105,106]. This marks a real shift: rather than simply copying nacre, sponge skeletons, or eggshells, investigators are pulling out the underlying design principles—staggered interfaces, directional reinforcement, crack deflection—and reimplementing them with synthetic building blocks and scalable processes [106,107,108]. Doing so makes biomimetic materials less tied to a specific natural template and easier to adapt to engineering constraints.

The most recent papers signal a move away from purely structural mimicry and toward functionally adaptive biomimetic materials. In biomedical applications, nanostructured biomaterials are now being designed for tissue regeneration, wound healing, biosensing, antimicrobial action, and drug delivery, frequently through smart interfaces or responsive scaffolds [109,110,111,112,113]. Nanocomposite GelMA bioinks and nanomaterial-integrated 3D-biofabricated structures, for instance, are being put forward as next-generation platforms because they improve mechanical robustness, conductivity, bioactivity, and stimuli-responsiveness while staying compatible with 3D printing workflows [114,115]. Similarly, scaffolds based on triply periodic minimal surfaces show how mathematically controlled geometries can reproduce porous biological functions and, at the same time, support manufacturability via additive manufacturing [116].

At the same time, the recent literature returns again and again to the same bottlenecks: scalability, reproducibility, interfacial stability, and safety [114,117,118,119,120]. Here is where biomimetic materials part ways with earlier bioinspired work. Older studies often demonstrated strong performance in small-format prototypes—ice-templated nacre analogues or biomimetic graphene aerogels, for example. Newer studies ask whether those architectures can be scaled, standardized, and folded into clinically or industrially relevant workflows [117,119,121]. The answer, so far, is a qualified yes, but only when fabrication is built around quality control, quantitative structure–property relationships, and process-aware manufacturing. Typical biomimetic material classes and their design logic are summarized in Table 2.

Biomimetic materials are best understood today not as straightforward replicas of nature, but as design frameworks for translating hierarchical biological principles into scalable, multifunctional materials [95,105]. The clearest recent trend is the coming together of nanofabrication, additive manufacturing, self-assembly, and quantitative design—a convergence that is making biomimetic structures more reproducible, more functional, and more directly relevant to biomedical translation than the single-scale mimics that came before [117,119,126,127].

## 4. Biomimetic Surfaces and Biomimetic Structures

Building on the material-level perspective, this section concentrates on biomimetic surfaces and thin functional structures. Biomimetic surfaces are engineered surfaces whose micro- and nanostructures draw on natural systems—the lotus leaf, shark skin, insect wings—and they display striking functional properties such as superhydrophobicity, antifouling, self-cleaning, and controlled adhesion by reproducing the hierarchical architectures found in those biological models. Biomimetic principles are harnessed here to achieve adaptability, comfort, and energy efficiency. What makes these surfaces significant is their capacity to translate biological design logic into advanced material performance, with impact across areas that range from biomedical implants to energy-absorbing structures and sensor platforms [128,129]. Despite these advances, the field has yet to converge on reliable structure–property design rules for many applications. Most performance data are collected under idealized single-contaminant or short-term assays, and it remains unclear how well laboratory-measured wettability or antibacterial efficiency translates to real-world conditions involving mechanical wear, mixed foulants, and long-term exposure. This gap fuels an ongoing debate about whether many of these surfaces are truly ready for deployment outside the lab.

Nanofabrication technologies have reshaped biomimetic surface engineering by offering precise control over structure and function across multiple length scales. Techniques like lithography, etching, deposition, and self-assembly make it possible to replicate complex natural architectures, while hybrid approaches that weave together top-down and bottom-up strategies deliver hierarchical ordering and multifunctionality [130,131]. These developments have moved biomimetic surfaces beyond laboratory prototypes and toward scalable, industrially relevant materials. Nevertheless, hybrid fabrication routes often trade simplicity for complexity, and the interfacial compatibility between soft organic templates and hard inorganic coatings remains a persistent weak point. A recurrent, unresolved issue is that gains in functional performance are frequently accompanied by losses in mechanical robustness or fabrication throughput, forcing compromises that are seldom systematically documented or compared across studies.

Hierarchical structures are a recurring architectural motif in biomimetic structural design. Hierarchy works by redistributing stress and strain, raising local stiffness, and encouraging stable deformation modes, all of which translate into better mechanical performance [132]. Hierarchical biomimetic designs—honeycombs and tubes, for example—markedly improve specific energy absorption (SEA), toughness, and load stability. Second-order structures can boost SEA over conventional designs [133,134], and dual-branching hierarchical square tubes along with bioinspired gradient multicell tubes show the largest SEA gains in recent studies [134,135]. Such hierarchical designs are central to achieving enhanced mechanical, antimicrobial, and multifunctional properties in biomimetic surfaces, with nanofabrication providing the means to control morphology and function with precision. A note of caution, however, is that most SEA values reported thus far come from quasi-static or low-strain-rate tests. Whether the same gains hold under dynamic, impact-relevant conditions is a matter of active debate.

At the micro- and nanoscale, surface topography reduces bacterial attachment and biofilm formation by limiting the available contact area and making detachment easier [136,137]. Combining microscale and nanoscale features strengthens both antifouling and antimicrobial performance, and sharper, denser features consistently outperform larger, more rounded ones [138,139]. Yet the mechanistic basis of this bactericidal effect remains contested. Moreover, almost all studies test single bacterial strains on freshly prepared surfaces; the long-term stability of the nanotopography and its antimicrobial action under repeated contamination, cleaning cycles, or protein-conditioning is rarely examined, leaving a substantial blind spot in the translation pathway.

Recent progress in nanofabrication and surface engineering has carried biomimetic surfaces into biomedical, sensing, and self-cleaning applications, with multifunctionality and adaptability standing out as emerging directions [136,140]. Surfaces inspired by the lotus leaf and shark skin, for instance, marry self-cleaning and antifouling properties in ways that support sensor platforms and wearable devices [131,139,140,141,142,143,144]. Antifouling coatings, in particular, are becoming critical for next-generation biosensors, where maintaining sensitivity and durability in complex environments is essential. Typical sources of biomimetic inspiration, fabrication methods, structural designs, and application areas are summarized in Table 3. It is worth noting that the bio-inspired branching hierarchical structures fabricated via additive manufacturing in Ref. [133] achieved a SEA increase of up to 282%, a figure that stands out markedly when compared with other recent biomimetic energy-absorbing designs. For instance, bio-inspired progressive gradient hierarchical quasi-honeycomb structures have been reported to yield a 17.4% SEA improvement [145]. Similarly, a corner-enhanced biomimetic spider web hierarchical structure exhibited a 46.3% increase in SEA [146], while a bionic variable cross-section tube achieved a 95.92% improvement relative to a circular tube [147]. Bioinspired tree-like fractal energy absorbers with a radial gradient design (TFGS-Pr) delivered a 41.5% rise in SEA [148]. These comparisons highlight the substantial advantage that the branching hierarchical architecture offers in terms of energy absorption efficiency, underscoring the promise of combining bio-inspired branching geometry with additive manufacturing for high-performance structures.

The current state of the art in biomimetic surface and structure design is marked by a strong push toward hybrid nanofabrication methods that combine top-down lithography with bottom-up self-assembly to produce highly functional, hierarchical structures. These advances open up new possibilities in biomedical implants, self-cleaning materials, and energy-absorbing systems, although challenges related to scalability, reproducibility, and long-term performance persist. Moving biomimetic surface nanofabrication from laboratory prototypes to industrial applications hinges on overcoming these very bottlenecks—scalability, reproducibility, and integration [126,128,155,156]. At the same time, a critical missing piece is a quantitative understanding of how hierarchical features degrade over operational lifetimes. Without such data, predictive design remains infeasible, and the field risks being guided more by trial-and-error than by engineering principle. At present, many biomimetic surfaces have not moved beyond the proof-of-concept stage; their long-term durability, cost-effectiveness, and real-world integration remain largely unvalidated, and some fabrication methods may not transfer easily across all material systems. An additional unresolved question is whether the remarkable performance seen in laboratory coupons can be replicated in complex geometries and large areas without loss of fidelity. Roll-to-roll nanoimprint and large-area colloidal lithography show promise, but they still struggle with defect control and pattern uniformity on curved or flexible substrates, a problem that becomes acute in wearable and implantable devices. Future work is expected to tackle these gaps through continuous-flow microfluidic synthesis, advanced characterization techniques, and the incorporation of artificial intelligence (AI) for process optimization [71,157,158,159].

## 5. Bio-Templating and Replication of Natural Structures

This section addresses bio-templating as a specific fabrication philosophy that uses natural structures directly as scaffolds or masters; it is therefore positioned as a bridge between the material and surface discussions and the device-oriented sections that follow. Bio-templating and replication have emerged as one of the most effective strategies for translating biological complexity into engineered materials, precisely because they can retain hierarchical morphology, interconnected porosity, and surface functionality that are difficult to achieve through conventional fabrication alone [160,161,162,163,164]. The underlying idea is straightforward: rather than attempting to invent a complex architecture from the ground up, researchers take a natural object, tissue, or biological scaffold as a ready-made template and then copy, invert, or reinforce its structure with a synthetic phase. Over the past decade, this has grown from a niche mimicry tactic into a broader manufacturing logic that now spans structural materials, catalysts, membranes, coatings, scaffolds, and energy devices [165]. The key change in recent work is that bio-templating is no longer judged solely by visual resemblance to nature; it is increasingly assessed on fidelity, scalability, functional retention, and process robustness.

### 5.1. Bio-Templating

**Figure 3:**
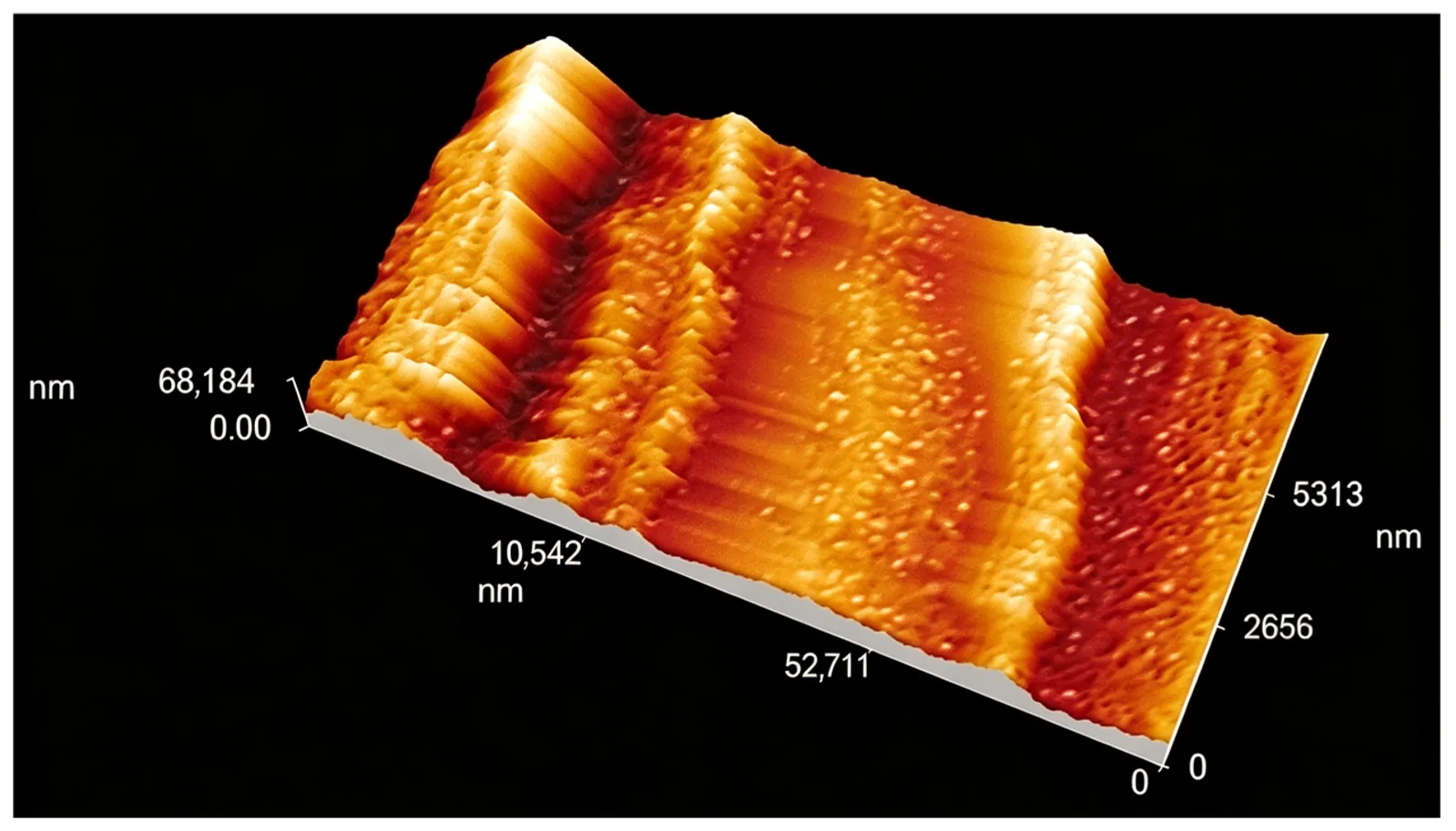
AFM image of biomimetic PDMS sample replicated from a bamboo leaf.

Bio-templating sets itself apart from ordinary pattern transfer because biological templates come with built-in multiscale order, from nanoscale roughness all the way to centimeter-scale vascular or lamellar organization [166,167,168,169]. This is why natural templates—leaves, wood, sponges, eggshell membranes, microbial scaffolds, decellularized tissues—keep reappearing as guides for replication [165]. Figure 3 shows the AFM image of the PDMS sample replicated from a bamboo leaf. The photo reveals that the replica is densely populated with needle-like fine protrusions, which exhibit considerable variation in height. Within the groove structures, numerous fine protrusions are densely distributed, substantially enhancing the surface roughness. Moreover, the ridge structures are composed of two or three narrower sub-ridges. These groove-and-ridge microstructures collectively suppress the ice formation rate and ice coverage, while also markedly inhibiting the retention of ice-melt water [170]. Unlike purely synthetic templates, these biological substrates often bundle chemistry and geometry into a single object, so the replicated product can inherit structural cues and surface chemistry together [166]. That dual inheritance carries weight in engineering because performance frequently depends on both: a rough surface may improve wetting or antifouling, for instance, but the same surface must stay mechanically stable under service conditions.

The field can usefully be read as a spectrum running from direct copying to functional reinterpretation. At the most literal end sits replica molding or nanocasting, where the template geometry is transferred into polymers, ceramics, or composites with high surface fidelity. A step beyond that is inverse replication, in which the natural template is removed and its void space becomes the final structure—a route seen in porous ceramics, carbon frameworks, and mineralized replicas. The newest direction is hybrid replication, where the template is not simply copied but chemically or mechanically upgraded, so that the final architecture keeps the biological blueprint while surpassing the original template in durability, conductivity, or corrosion resistance. This marks the most important conceptual advance in recent studies: biomimicry is shifting from imitation toward performance translation [171].

A less discussed but important tension is that template complexity and template removal often work against each other: the more delicate and interconnected the biological architecture, the more likely the removal step will damage the replica. For closed-cell or highly entwined structures, achieving complete template extraction without crack formation or feature collapse remains an unsolved processing challenge, and this practical hurdle is one reason why many bio-templated materials are still limited to open-pore architectures.

### 5.2. Major Biological Templates

Natural templates are not interchangeable; each class offers a different structural advantage. Plant surfaces such as lotus leaves, rose petals, goose feathers, and Xanthosoma leaves lend themselves well to surface replication because they carry well-defined micro- and nanotextures linked to superhydrophobicity, self-cleaning, antifouling, and anti-corrosion functions. These systems are attractive when the goal is a functional coating rather than a bulk scaffold. Wood and loofah, by contrast, are frequently used as macroscopic templates for porous ceramics and thermal-management structures, since they naturally contain aligned channels and interconnected voids that are hard to produce synthetically at scale. Sponges and eggshell membranes matter for similar reasons: their reticulated networks supply a ready-made architecture for hierarchical ceramics and catalytically active supports.

At the microstructural end of the spectrum, microbes, viruses, bacteria, fungi, algae, and bacterial cellulose are valuable because they function as biologically programmable templates with reproducible nanoscale organization and rich surface chemistry [165,166]. These templates prove especially effective when the aim is to create nanostructured materials with controlled chemistry, not just copied shape. Earlier reviews on microbes as structural templates have already shown that microbes can bridge nano- to mesoscales through bottom-up routes [166], and more recent work on bacterial cellulose extends that idea into living, adaptive fabrication platforms [165]. Between these extremes lie natural tissues such as bone, dentin, cartilage, and enthesis, which are used when the goal is to replicate both structure and mechanical function [172]. Here, the challenge goes beyond copying porosity: it involves reproducing zonal transitions, mineral gradients, and load-transfer pathways.

### 5.3. Fabrication Routes

The dominant fabrication logic in bio-templating remains template impregnation followed by conversion or removal, but the choice of route strongly shapes fidelity and properties. In direct replication, a polymer or sol–gel precursor is cast onto a biological surface—leaf, rose petal, feather—and then cured to reproduce topography. This route is fast and practical for functional surfaces, though it is usually limited to shallow or moderate relief and can struggle with undercuts or fragile templates.

In foam replication or sponge replication, a porous biological template is impregnated with a ceramic or precursor slurry and later burned out or converted, yielding open-cell structures that echo trabecular bone or reticulated foams. The strength of this approach lies in its ability to generate bulk architectures rather than just surface textures, and its value for load-bearing scaffolds and high-temperature ceramics is well recognized. Ice templating (freeze casting) belongs to the same conceptual family: here the biological template is sometimes replaced by ice as a transient template, but the method still reproduces the aligned, hierarchical pore logic observed in natural tissues and woods [173]. Compared with direct biological replication, freeze casting offers greater compositional freedom and scale-up potential, though it does not preserve template-specific surface chemistry.

The most recent trend is hybrid bio-templating, where template copying is paired with secondary treatments such as atomic layer deposition, glancing-angle deposition, photopolymerization, mineralization, or multi-material printing. This development is important because many biological templates are mechanically weak, chemically unstable, or too variable for direct end use [165]. Hybrid workflows address these shortcomings by using the template only as a structural guide and then converting the replicated form into a more robust engineering material. The trade-off, however, is that each added processing step multiplies the sources of variability and cost, making it harder to isolate which step dominates the final property envelope. Very few studies provide a breakdown of how much performance gain can be attributed to the template structure versus the post-treatment, which clouds the picture for anyone attempting to optimize a similar route.

### 5.4. Structure–Property Relationships

Replication is only useful if the copied structure preserves the function-carrying features of the original biological system. In many cases, the replicated material works because the template encodes hierarchical roughness, anisotropic transport channels, or load-bearing architectures that directly govern performance. Lotus- and rose-inspired textures, for example, improve hydrophobicity and antifouling through combined micro- and nanoscale roughness, while feather-inspired geometries enhance corrosion resistance by promoting water repellence and reducing biofilm attachment. In thermal and structural materials, wood-, horn-, and bone-inspired templates improve strength-to-weight ratio, thermal insulation, or energy absorption because aligned pores and lamellae guide crack deflection and stress redistribution.

A critical point is that fidelity alone is not enough. Directly copied natural structures may reproduce morphology well yet miss the full interface mechanics or crystallographic features of the source material. The replica can look biologically authentic and still underperform if the chemistry, phase distribution, or interfacial bonding is not properly tuned. This explains why recent bio-templating research increasingly couples structural copying with compositional design—mineral doping, polymer infiltration, scaffold reinforcement [160,172]. The practical takeaway is that the best biomimetic replicas are usually structure-plus-material systems, not pure morphology copies [171,174].

What remains largely absent from the literature is a head-to-head comparison of a bio-templated replica with the original biological material under identical test conditions. Such comparisons are rare because living tissues are adaptive and hierarchically more complex, but without them the claim of “bio-fidelity” often rests on visual similarity rather than on functional equivalence. Furthermore, the long-term environmental stability of bio-templated materials—under humidity, thermal cycling, or repeated loading—is seldom reported, even though it is precisely these conditions that will determine whether a templated material can move from the laboratory to a real application. Addressing these gaps will require not only better characterization but also a more critical and transparent reporting culture, one that acknowledges when replication fails to capture the functional essence of the biological model.

## 6. Applications of Nanofabrication in Bionic Devices and Systems

Whereas the preceding sections center on materials and surfaces, this section shifts the focus to integrated devices and systems, covering selected application domains where nanofabricated biointerfaces drive functional performance at the system level. Nanofabrication has moved to the center of bionic device and system development because it gives engineers control over surface chemistry, topography, and multiscale architecture in ways that capture biological function more faithfully than bulk materials alone. Over the past decade, the emphasis has shifted from simply miniaturizing devices toward engineering biointerfaces that actively regulate cells, fluids, signals, and mechanics [143,175]. As a result, nanofabrication now reaches well beyond biosensors and drug delivery into organ-on-chip platforms, biohybrid tissues, wearable electronics, and soft robotic systems [112,144,176].

### 6.1. Biomimetic Biosensors and Bioelectronic Interfaces

Earlier biosensor designs tended to optimize for single-analyte readout, whereas newer biomimetic platforms increasingly bring together multimodal sensing, microfluidic compatibility, and flexible substrates [111,112,144]. This shift reflects a practical reality: biological interfaces rarely handle one cue in isolation—they process multiple signals at once, often under dynamic mechanical conditions. Nanofabrication supports this transition by enabling controlled patterning of materials and structures at the very scale where recognition events take place, which in turn improves analytical performance and opens the door to continuous monitoring rather than episodic testing [111,143].

Recent work also points to a stronger convergence between biosensing and biomedical deployment. Wearable and biointegrated devices are being designed more and more for real-time health monitoring, point-of-care diagnostics, and precision medicine, signaling a move from laboratory prototypes toward systems that must perform in practical settings [118,143]. At the same time, the literature consistently shows that performance gains still hinge on fabrication quality, interface stability, and standardization—not simply on which nanomaterial is chosen [111,121,143]. Put differently, the field’s challenge has shifted from demonstrating sensitivity to building reproducible biomimetic bioelectronics.

### 6.2. Drug Delivery and Controlled Release Systems

Nanofabrication has reshaped bionic drug delivery by offering precise control over geometry, payload loading, and release kinetics. Reviews from 2016 and 2026 highlight that micro- and nanoscale fabrication can produce microneedles, reservoirs, carriers, and stimuli-responsive systems with an architectural precision that conventional formulation routes cannot match. From a biomimetic standpoint, this matters because biological transport systems do not rely on simple bulk diffusion; they depend on structured pathways, local compartmentalization, and regulated release.

**Figure 4:**
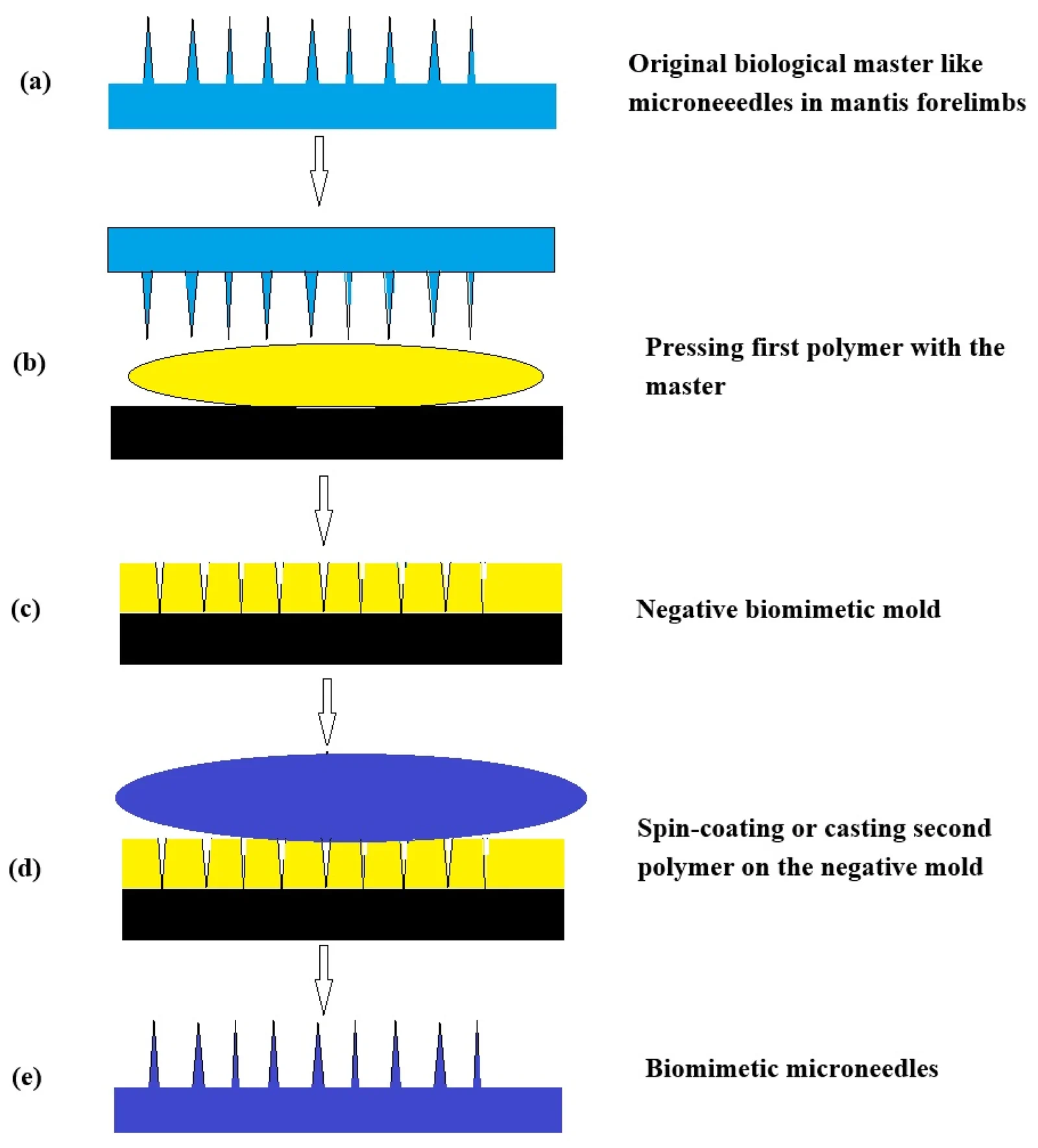
Fabrication processes of bionic microneedles (blue represents the master, yellow represents the first polymer, black represents the substrate, and purple represents the second polymer). (**a**) original biological master; (**b**) pressing first polymer with the master; (**c**) negative biomimetic mold; (**d**) spin-coating or casting second polymer on the negative mold; (**e**) bionic microneedles.

A notable trend is the move away from microfabrication as a tool for isolated delivery structures and toward integrated platforms that combine sensing, actuation, and release [5,177,178]. Microneedles are a representative case: they reduce invasiveness while preserving delivery efficiency, and newer fabrication strategies improve both design flexibility and manufacturability [5,179]. Figure 4 illustrates the fabrication process of bionic microneedles. A bioinspired master was first fabricated using natural templates such as mantis forelimbs. Nanoimprint was then used to transfer the master pattern onto a polymer substrate, and upon demolding a negative replica was obtained. A second polymer layer was deposited onto the negative mold via spin-coating or casting, and finally bionic microneedles were successfully fabricated.

Compared with traditional drug delivery formats, nanofabricated systems are better able to reproduce the spatial and temporal precision of native physiological release. That precision is especially valuable when local dose, release timing, and tissue contact all influence therapeutic outcome—because nanofabrication allows these parameters to be encoded directly into device architecture rather than adjusted only through chemistry [5,178]. In this sense, nanofabrication moves drug delivery from formulation-centered design toward structure-guided design.

Recent reviews increasingly treat drug delivery as a systems problem rather than a single-device problem. From this perspective, nanofabrication is valuable because it makes it possible to engineer the drug, carrier, and target tissue as a coupled unit, which more closely mirrors how biological delivery pathways actually work [5,178]. The same literature also stresses that translational progress depends on reproducibility, long-term stability, and regulatory validation—meaning that architectural precision must be matched by manufacturable robustness [5,179].

Taken together, the biomimetic value of nanofabrication in drug delivery rests on its ability to translate biological principles—compartmentalization, responsiveness, localized transport—into engineered platforms. The field’s most important direction is therefore not simply smaller carriers or finer features, but more integrated and reproducible release systems that can operate with the same selectivity and efficiency as natural delivery pathways [5,178,179].

### 6.3. Tissue Engineering and Biofabricated Bionic Systems

In tissue engineering, nanofabrication is at its most powerful when it creates cell-instructive microenvironments rather than merely shaping scaffolds. Recent reviews show that nanomaterials incorporated into 3D-biofabricated structures can improve mechanical strength, conductivity, bioactivity, and drug delivery, supporting applications in nerve, bone, cardiac, and wound repair. Compared with earlier scaffold-centered work that prioritized porosity and form, the current direction leans toward multifunctional constructs that regulate cells through both architecture and composition [114,115,126].

Single-material constructs are simpler to fabricate, but hybrid biofabricated platforms come closer to the hierarchical organization of native tissues because they can integrate gradients, interfaces, and nanomaterial-enabled functionality [114,180,181]. Reviews on process hybridization and multiscale engineered tissue biofabrication make one point clear: no single process can fully reproduce the nano-to-macro hierarchy of living tissue, which is why hybrid fabrication has gained ground [127,180].

Recent studies also reveal a shift from structural scaffolds toward functional tissue platforms. Nanocomposite GelMA bioinks and nanomaterial-integrated 3D systems are now put forward as next-generation tools because they improve printability while adding conductivity, bioactivity, or stimuli responsiveness [114,115]. This trend is biomimetic in a deeper sense: native tissues are not simply shaped structures but dynamic systems that couple mechanics, signaling, and metabolism. Still, the literature repeatedly flags unresolved issues around interface quality, reproducibility, and scale-up [114,115,180].

### 6.4. Organ-on-Chip and Lab-on-Chip Systems

Organ-on-chip systems stand among the clearest bionic applications of nanofabrication because they recreate flow, compartmentalization, and cell organization within controlled microenvironments [176,182,183]. Reviews on organ-on-chip and lab-on-chip platforms demonstrate that microengineering and 3D biofabrication can reproduce physiological architecture more faithfully than 2D culture, improving both disease modeling and drug screening [176,182,184]. Here, nanofabrication is not simply a manufacturing method; it is what makes biomimetic function experimentally controllable.

A useful contrast can be drawn between simple microfluidic chips and more elaborate tissue-specific bionic chips. Simple chips have the advantage of being compact and reproducible, but they often oversimplify biology; more advanced platforms improve physiological relevance yet increase fabrication difficulty and protocol complexity. Reviews on gut-on-chip and bone-on-chip technologies indicate that the field is moving toward richer models with better tissue-specific organization and integrated sensing, while also acknowledging that standardized benchmarks are still lacking [184,185].

A broader pattern is that chip-based systems are increasingly deployed as interfaces between biomaterials and biology rather than as isolated devices. The recent interface-centered literature stresses that fabrication strategy, material selection, and biological function must be designed together if the chip is to function as a realistic bionic platform [175,176,183]. This places nanofabrication at the center of organ-on-chip development, because it enables both the physical architecture and the functional interface required for realistic modeling.

### 6.5. Soft Robotics, Wearable Systems, and Biohybrid Devices

Soft robotics and biohybrid devices lean heavily on nanofabrication because biomimetic motion demands compliance, distributed sensing, and multiscale integration [112,186,187,188,189]. Recent reviews underscore that nanomaterials and nanofabrication help produce soft actuators, flexible sensors, and integrated interfaces for biomedical and wearable applications [112,190,191]. Compared with rigid robotics, these systems are inherently more biomimetic: they approximate the softness, adaptability, and mechanical coupling observed in living organisms [187,188].

The field has clearly progressed from motion-only prototypes toward motion plus sensing plus functional integration. Earlier work concentrated on getting small soft robots to move, whereas more recent studies emphasize embedded functionality, conformable piezoelectric systems, and biomimetic intelligence [186,187,188,189,190,191,192]. This shift is significant because it brings robotic design into closer alignment with biology, where locomotion, sensing, and response are inseparable rather than modular.

Biohybrid robotics pushes this trajectory further by incorporating living tissue or cell-powered actuation into engineered structures. These systems are especially biomimetic because they borrow both material behavior and actuation principles from living systems, yet they also face the highest barriers in reproducibility, durability, and long-term stability [193,194,195]. The literature therefore points to a clear conclusion: nanofabrication is enabling increasingly lifelike soft systems, but practical translation hinges on robust manufacturing and stable operation under real physiological conditions [112,187,188,189,194].

The main application areas are listed in Table 4. Across these five directions, nanofabrication carries the most weight when it translates biological function into manufacturable architecture rather than merely shrinking device dimensions [5,112,175,176]. The recent trend is the move from isolated components to integrated systems that combine structure, transport, signaling, mechanics, and feedback [114,118,143,186]. That makes nanofabrication not just a tool for fabricating bionic devices, but a design framework for building bionic systems.

## 7. Cross-Scale Biomimetics: System Integration from Nanoscale to Macroscale

This section synthesizes the earlier themes and explicitly articulates the cross-scale design logic. Cross-scale biomimetics is emerging as the central design logic for biomimetic structures, largely because biological performance arises not from any single feature but from the way nanoscale chemistry, microscale organization, and macroscale geometry work together [89,95,196]. In engineering terms, the aim is no longer to copy a natural shape; it is to reproduce the coupling rules that link interfaces, hierarchies, and load transfer across length scales. This explains why recent work increasingly treats multiscale design as a systems problem: one material must simultaneously handle strength, toughness, transport, responsiveness, and manufacturability [105,197,198,199,200]. Despite this conceptual consensus, the field still lacks a set of quantitative, generalizable coupling rules. Most successful cross-scale systems are discovered through intuition-driven trial and error, and it remains unclear under what conditions a particular combination of nano- and microscale features will yield synergistic rather than antagonistic effects.

A guiding principle in this area is that nanoscale features typically supply the local mechanism, while macroscale architecture sets the system-level function. Single-scale approaches can deliver useful effects—superhydrophobicity, local stiffness tuning, enhanced surface interactions—but they tend to plateau because the remaining scales are not coordinated. Nanoscale interfaces, for instance, can improve adhesion, crack deflection, or chemical responsiveness, yet those gains often remain partially expressed unless they are organized into layered, aligned, or gradient macroscale structures [105,196]. This vertical relationship mirrors what is seen in biological materials, where mineralized collagen fibrils, Bouligand order, or porous ceramics draw their full value from scale bridging rather than from isolated nanoscale reinforcement [196,201,202,203,204,205]. By comparison, cross-scale biomimetics can weave together microscopic energy dissipation, mesoscale crack control, and macroscale shape guidance within a single material system, which is why recent hierarchical composites and metamaterials strike a better balance among properties that would otherwise conflict [101,199,206,207,208,209,210].

Processing techniques for cross-scale biomimetics can be grouped into two categories: top-down deterministic manufacturing and bottom-up hierarchical assembly [101,202]. Top-down methods such as 3D printing and direct patterning excel at macroscale geometry and repeatability, but they frequently require nanoscale correction afterward. Bottom-up routes, by contrast, are better at generating fine order and multifunctional interfaces, yet they struggle with geometric freedom and large-size fabrication. Current work increasingly merges the two—for example, by printing a macroframework and then letting self-assembly, crystallization, or infiltration generate the finer hierarchy [101,126,202,211]. Earlier approaches often used one method to produce one scale and then accepted a mismatch with the remaining structure [212,213]. Newer strategies deliberately combine self-assembly, 3D printing, microfluidics, template-assisted synthesis, and surface modification so that each process addresses a different length scale [126,202,211,214]. This hybrid logic matters because it turns multiscale architecture from a processing accident into a controllable design outcome. Yet, hybrid fabrication also multiplies the sources of variability. Each processing step introduces its own tolerances and defect populations, and how these defects propagate across scales—and whether the final property envelope is dominated by the coarsest or the finest feature set—is not well understood. Moreover, compatibility between disparate process chemistries remains a persistent practical obstacle; an optimal self-assembly condition for the nanoscale may be incompatible with the curing or sintering cycle required by the macroscale framework, forcing compromises that are seldom documented transparently.

Recent studies also point to interface engineering as the real bridge between scales [105,197,215]. In biohybrid composites, nano-, micro-, and macroscale interfaces can each contribute a distinct function: nanoscale chemistry governs bonding, microscale architecture governs stress redistribution, and macroscale topology governs structural integration with the surrounding environment [203]. This becomes especially clear in gradient systems, where smooth transitions avoid abrupt mechanical mismatch and improve load transfer, much like the enthesis and other natural transition zones. In this sense, biomimetics is moving away from “surface decoration” and toward “hierarchical interface choreography” [105,197]. However, experimental characterization of interfacial mechanical and chemical states across three length scales remains a formidable challenge, especially under in situ or operando conditions. As a result, many interface-engineering strategies are designed on the basis of idealized assumptions rather than direct, scale-resolved measurements, and the correlation between interfacial design and system-level durability is often inferred rather than demonstrated.

Mechanically, cross-scale design is valuable because it helps resolve long-standing trade-offs: stiffness versus toughness, strength versus porosity, lightness versus damage tolerance. Hierarchical materials can deflect cracks, localize deformation, and distribute stress in ways that simple homogeneous structures cannot. This is evident in brick-and-mortar systems, Bouligand architectures, and layered nanocomposites, where microscale arrangement often matters as much as constituent chemistry [202,210,216]. The main takeaway is that mechanical performance in biomimetic materials is not additive across scales; it emerges from their interaction.

The recent literature places growing weight on AI, quantitative biomimetics, and multiscale modeling [95,105,217]. Rather than relying solely on qualitative imitation, newer studies attempt to link geometry, interfacial chemistry, and process variables to properties through predictive frameworks [218]. This is important because scale bridging is inherently complex: nanoscale ordering may improve local interactions, but that same ordering can be lost during bulk consolidation unless the model captures the full chain from assembly to architecture. The emerging direction is therefore “design by translation,” where biological principles are converted mathematically or computationally into manufacturable structures [105,217].

**Figure 5:**
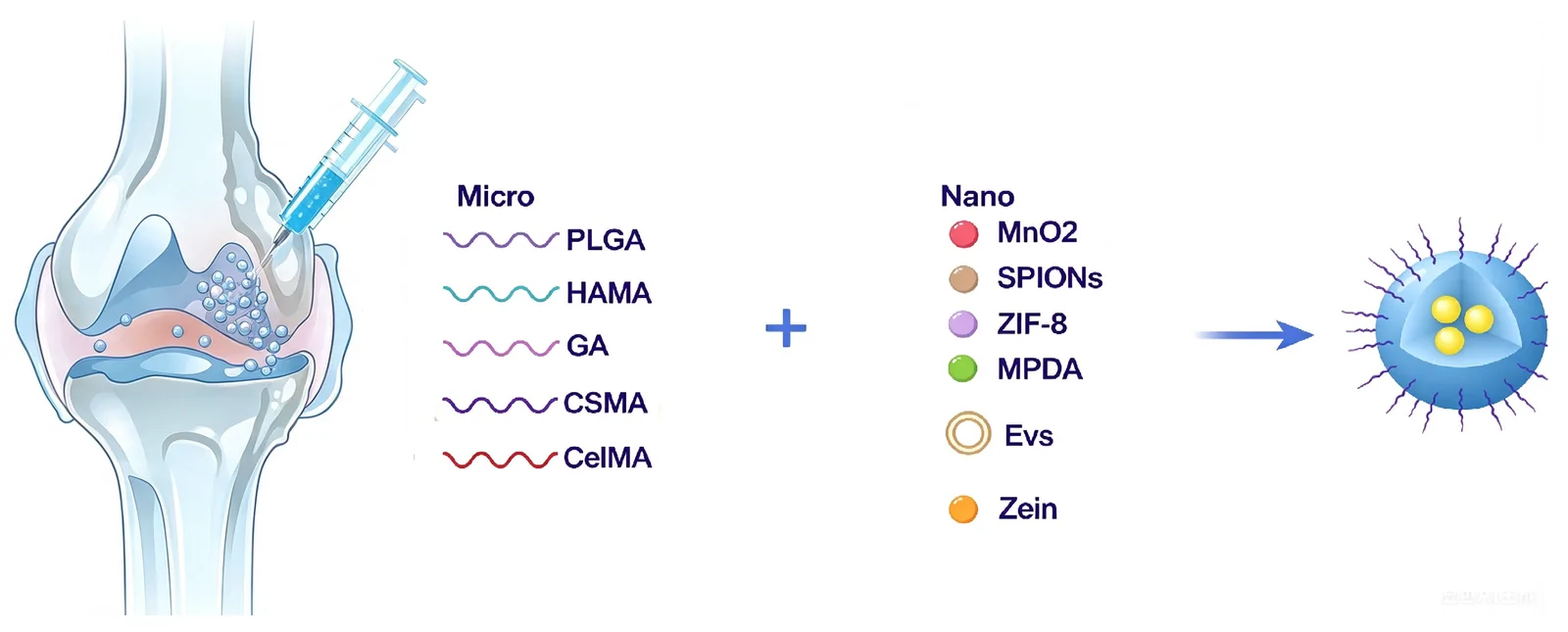
Schematic illustration of the conceptual framework of micro-nano composite architectures for integrated osteoarthritis (OA) theranostics. This hierarchical design paradigm synergistically integrates diverse micrometer-scale matrices (represented by PLGA, HAMA, GA, CSMA and CelMA) with functional nanounits (including MnO_2_, superparamagnetic iron oxide nanoparticles (SPIONs), zeolitic imidazolate framework-8 (ZIF-8), Mesoporous Polydopamine (MPDA), extracellular vesicles (EVs) and Zein), to achieve coordinated biophysical modulation and biochemical signaling crosstalk for enhanced therapeutic outcomes. The composite system is delivered into the joint cavity via intra-articular injection. The microscale framework ensures prolonged retention within the joint, while the released nanoscale units facilitate deep penetration into the extracellular matrix and subsequent intracellular drug exposure [222].

Cross-scale biomimetics is also gaining ground in functional systems beyond mechanics, most notably in thermal management, wetting, and transport [219,220,221,222]. Nanoscale texture alone is seldom sufficient; it must be embedded within larger directional pathways or multilayer stacks to generate robust macroscopic performance. Leaf-vein-like thermal composites, liquid-infused fog-harvesting frames, and layered thermomechanical composites all illustrate that structural hierarchy can guide heat, liquid, and stress in a controlled manner. By recapitulating the heterogeneous architecture of native articular cartilage, the systems designed by Shi et al. can construct a biomimetic niche that is well-suited for tissue repair, as shown in Figure 5 [222]. Specifically, the micrometer-scale frameworks with precisely controlled pore sizes ranging from 20 to 100 μm can emulate the spatial arrangement of collagen fibers, providing physical anchoring sites for cells while optimizing the internal diffusion efficiency of nutrients. Meanwhile, nanoscale functional units can accurately mimic the topological features of the extracellular matrix (ECM) to further enhance intracellular delivery efficiency. This hierarchical design, which often incorporates biphasic structures, enables the mechanical parameters of the system, such as compressive modulus, to be regulated above 50 kPa, achieving mechanical property matching with native cartilage. Most critically, recent research advances have shifted from the early-stage static stiffness matching to the direction of dynamic viscoelasticity recapitulation: by introducing dynamic covalent bonds (e.g., Schiff base bonds or hydrazine bonds) into the micro-matrix, the stress relaxation time constant of the micro-nano platform can be made comparable to that of native tissue. This “stress-relaxing” property allows the carrier to dissipate external mechanical energy, preventing destructive impact stress from being directly transmitted to the chondrocytes residing within the cartilage.

The recurring pattern is that microscale patterning produces the local effect, while macroscale organization makes that effect useful in actual devices. Recent work further shows that cross-scale biomimetics is shifting toward device integration, not just material synthesis [223,224,225,226]. Taking cues from natural nacre, biomimetic design principles resolve the long-standing challenges of MXene brittleness and van der Waals-induced nanosheet restacking. The Song team presented a hierarchical modification strategy framework that integrates three distinct levels: intrinsic functionalization of individual MXene nanosheets, interfacial engineering within composite systems, and macroscale structural architecture [223], through which flexible MXene actuators can be produced. This multi-level approach is specifically designed to modulate mismatch efficacy, whereby the macroscale architectural strategies serve as the critical bridge that faithfully translates microscopic physicochemical properties into precisely controllable macroscopic mechanical work.

In biosensors, actuators, and optical devices, functional performance hinges on how nanoscale sensing elements, mesoscale pathways, and macroscale form are combined into one system. This marks a move from “make the structure” to “make the structure work as a system”—a more mature biomimetic agenda [95,200,227]. In this respect, cross-scale biomimetics is increasingly converging with smart materials and biohybrid devices rather than remaining a purely structural materials topic. This convergence, while promising, also creates new reliability challenges: a smart material that changes its properties in response to environmental stimuli must maintain its cross-scale hierarchy across repeated actuation cycles, which introduces fatigue and aging mechanisms that are poorly understood at the nanoscale. The field has barely begun to address how mechanical cycling affects the hierarchical integrity of such systems.

This scale-combination strategy has proven especially effective in porous and lightweight systems [199,205,213]. Hierarchically porous ceramics, for example, use pore size distribution to separate functions: nano- and mesopores can aid surface reactions or bioactivity, while macropores support transport and structural continuity. The same logic appears in wood-, sponge-, and shell-inspired structures, where architecture rather than composition drives the balance among stiffness, permeability, and weight [228,229,230,231]. In these materials, multiscale porosity is not a defect; it is the design principle that makes the material usable. Despite this, achieving simultaneous optimization of stiffness, permeability, and weight across a broad porosity range remains non-trivial. There is usually a trade-off: increasing interconnected porosity to improve transport often compromises strength, and the optimal pore size distribution for one function (e.g., nutrient diffusion) may be suboptimal for another (e.g., mechanical load bearing). Quantitative design maps that reconcile these competing demands are conspicuously rare.

The main bottleneck remains “scale-up without losing hierarchy” [198,215,227,232]. Recent reviews repeatedly note that laboratory demonstrations often succeed because they tightly control structure at one or two scales, but industrial deployment becomes difficult when uniformity, throughput, and defect control must all be maintained at once [198,232,233,234]. This is why scalable approaches—microfluidic fabrication, additive manufacturing, roll-to-roll processing, solvent-free hybrid coatings—are drawing so much attention. The field’s practical challenge is not simply making hierarchy; it is making hierarchy reproducibly, at size, and with retained function.

Taken as a whole, cross-scale biomimetics is best understood as an engineering strategy for turning biological complexity into manufacturable function. Its progress is being driven by the convergence of multiscale modeling, interface engineering, hybrid fabrication, and quantitative design. The most successful systems are those that align local chemistry, intermediate architecture, and global geometry into one coherent hierarchy. That is why the field is moving away from isolated imitation and toward “integrated multiscale translation,” where the real target is not nature’s form, but nature’s organizing principle [89,95,196,202].

## 8. Challenges and Future Directions

Nanofabrication has made biomimetic structures considerably more sophisticated, yet the field is now constrained less by whether biomimetic architectures can work than by whether they can be produced reproducibly, safely, and at scale. Recent reviews return to the same bottlenecks: interfacial instability, scalability, manufacturing reproducibility, biosafety, and regulatory readiness continue to block translation, even when proof-of-concept performance is strong [114,197,235,236,237]. This is particularly evident in hybrid and multiscale systems, where functional gains often rest on delicate coupling across nano-, micro-, and macrointerfaces—the very interfaces most vulnerable to phase separation, aggregation, delamination, and performance drift [238,239,240,241]. Put simply, the central challenge is no longer design inspiration, but engineering control.

One major technical barrier is the “resolution-versus-scalability” tension. High-resolution methods—electron-beam lithography, nanoimprint, femtosecond laser patterning, two-photon polymerization, and other precision routes—can reproduce fine biomimetic features, but they tend to struggle with throughput and cost when the target is a clinically or industrially relevant area [242,243,244,245,246]. Additive manufacturing and hybrid biofabrication, by contrast, are far stronger on volume and geometry, yet they still face resolution limits and often cannot fully recapitulate native multiscale complexity on their own [127,247,248,249]. The practical path forward is therefore not to pick one platform over another, but to combine them in “hybrid process chains” that assign each method to the scale it handles best [126,243,249,250].

A second challenge is “interface stability under real operating conditions”. Multiple recent reviews stress that biomimetic performance frequently degrades when temperature, humidity, chemical exposure, mechanical loading, or biological fluids alter the engineered interface [235,239,240,251]. This problem cuts across material classes: it shows up in biohybrid composites, implant coatings, nanomaterial-integrated scaffolds, and biomimetic delivery systems alike [197,236,252]. The direction forward lies in “interfacial engineering with built-in durability”—graded layers, hierarchical confinement, stronger yet biologically compatible coupling chemistries, and stimulus-responsive interfaces that preserve function while adapting to their environment [197,241,251,253].

A third bottleneck is “biosafety and life-cycle behavior”. For biomedical biomimetic systems, the literature increasingly cautions that long-term fate, biodegradation, immune response, environmental release, and degradation products must be treated as design variables, not as afterthoughts [240,254,255,256]. Reviews on nanobiomaterials and nanomedicine underscore the shortage of standardized analytical characterization, standardized biological assays, and reliable long-term safety evaluation [121,257,258]. Future work should therefore move toward safe-by-design biomimetic nanofabrication, supported by standardized characterization workflows, validated in situ or ex situ testing, and clearer links between structure, exposure, and biological outcome.

The most promising direction is “quantitative, data-driven biomimetics”. Recent perspective and review papers argue that the field must go beyond qualitative imitation and toward predictive design frameworks that connect geometry, interfacial chemistry, and performance [95,105,259,260]. This includes multiscale modeling, digital twins, machine learning (ML), and in situ characterization aimed at mapping how nanoscale changes propagate to system-level function [203,259,260,261]. A key inference from these studies is that future biomimetic materials will be designed less by copying a structure and more by optimizing a structure–function landscape. Recent reviews show that AI is shifting biomimetic materials design away from trial-and-error toward data-driven inverse design, structural optimization, generative morphogenesis, and closed-loop discovery [262,263]. In biomimetic lattices and metamaterials, ML-based design optimization is already being used to accelerate exploration of high-dimensional design spaces and improve structure–property relationships [216,264,265,266]. A useful framing is that AI is not replacing biomimicry, but making biomimetic design more searchable, faster, and more scalable.

Sustainable biofabrication is increasingly tied to biocompatibility, biodegradability, green reagents, renewable feedstocks, and reduced waste generation. Recent work also highlights eco-friendly fabrication routes such as biomimetic synthesis, electrospinning, additive manufacturing, and micro/nanofabrication using natural-origin materials like cellulose, chitosan, pectin, lignin, and related biopolymers [258,267,268,269,270]. For biomedical use, the practical advantage is that degradable systems can reduce the need for removal surgery and support transient-function implants.

For biomimetic medical devices, the literature consistently emphasizes risk-based classification, biocompatibility testing, quality systems, preclinical validation, and post-market surveillance as core regulatory elements [271,272,273]. Reviews on 3D-printed and personalized devices further note that global regulatory divergence remains a major barrier, especially when devices combine complex structures, customized manufacturing, and novel materials [274,275,276]. Recent discussions also point to emerging tools such as digital twins, in silico trials, and AI/ML-enabled assessment, but these are still framed as developing pathways rather than fully harmonized standards [277,278,279].

A practical dimension that remains underrepresented in the biomimetic nanofabrication literature is the regulatory pathway. Most biomimetic surfaces and devices intended for clinical use will, depending on their claimed mechanism of action, fall under medical device or combination-product regulations and must navigate either the FDA’s 510 (k) or PMA (Premarket Approval) routes, or the EU’s MDR (Medical Device Regulation) with CE marking via notified bodies. The challenge is that hierarchical, bioinspired nanostructures often do not fit neatly into existing product categories. A nano-patterned antibacterial surface, for instance, may act physically rather than chemically, raising questions about how to classify it and which biocompatibility standards apply. Quality control is equally problematic: the critical performance attribute is frequently the nanostructure itself, yet standardized methods for routine, non-destructive inspection of nanoscale topography across large areas are still under development.

In summary, the field is moving toward biomimetic structures that are hybrid, scalable, quantitatively designed, and regulation-ready, rather than merely visually nature-like. The strongest future systems will likely combine precision nanofabrication with additive manufacturing, multiscale interface control, AI-driven, sustainable and biodegradable fabricating, and standardized safety validation, so that biological inspiration becomes a manufacturable engineering platform rather than a laboratory demonstration.

## 9. Conclusions

Nanofabrication has become the enabling toolkit that moves biomimetics from inspiration to implementation. The most powerful direction of development is the convergence of bottom-up assembly, top-down patterning, bio-templating, and process hybridization—a combination that makes it possible to construct bionic systems across length scales while keeping their functional relevance intact. This trend is particularly visible in recent work on hierarchical composites, multifunctional interfaces, biofabricated tissues, and integrated devices. Looking ahead, the central question is not whether biomimetic structures can be fabricated, but whether they can be fabricated reproducibly, at scale, and with stable performance. Future progress will most likely turn on quantitative design, standardized testing, and manufacturing strategies that preserve hierarchy during scale-up. In this sense, the field is shifting toward integrated multiscale translation, where the aim is no longer to imitate nature’s form, but to reproduce nature’s organizing principles in practical engineered systems. As nanofabrication techniques keep advancing, their application in the preparation of biomimetic structures can be expected to widen, offering new solutions to challenges in biomedicine, sensing, energy, and beyond.

## Figures and Tables

**Table 1:** Comparative evaluation of major nanofabrication techniques for biomimetic structures.

Technique	Typical Resolution	Throughput	Relative Cost	Material Compatibility	Biomimetic Suitability	Key Limitations for Biomimetics
Photolithography	5 nm–1 µm	Very high (wafer-scale)	High (cleanroom)	Photoresists, Si, glass	Regular arrays, microfluidics, cell-culture platforms	2.5D only; cannot produce undercuts or true 3D porosity
Nanoimprint lithography	~10 nm	High	Medium (mould cost)	Thermal/UV polymers	Replication of natural surface textures (shark skin, lotus leaf)	Mould wear, demoulding defects; poor 3D structural control
Soft lithography	~30 nm	Medium	Low	PDMS, biomolecules, hydrogels	Microfluidic networks, organ-on-chip, cell alignment	Mechanical deformation; limited aspect ratio
Electron=beam lithography	Sub-10 nm	Very low	Very high	Electron resists	Master templates, protrusive nanostructures (moth-eye)	Serial writing, impossible to scale up to cm^2^
Focused ion beam	Sub-10 nm	Very low	Very high	Wide (metals, ceramics, polymers)	Nano-prototyping, TEM sample preparation	Sample damage, Ga contamination, miniscule throughput
Two-photon polymerization (2PP)	~100 nm	Low	Medium-high	Photosensitive resins	True 3D lattices, trabecular scaffolds, microlenses	Slow build speed, limited resin choice
Electrospinning	Fibre diameter ~10 nm–5 µm	High (continuous)	Low	Many polymer solutions	Extracellular matrix-like fibrous mats	Fibre orientation hard to control; 3D shaping limited
Molecular self-assembly/DNA origami	~1–5 nm	Medium (batch)	Medium (DNA expensive)	Custom molecules, DNA	Lipid bilayers, molecular drug carriers, photonic crystals	Long-range order difficult; scaling to macroscopic devices unresolved
Colloidal lithography	~50 nm (sphere size dependent)	High (self-assembly)	Low	Polymers, metals on substrates	Anti-reflective coatings, plasmonic arrays	Defects in colloidal crystal; limited to closed-packed geometry

**Table 2:** Typical biomimetic material classes and their design logic.

Material Class	Main Biomimetic Logic	Distinct Advantage	Main Limitation
Layered and nacre-like composites	Brick-and-mortar architecture, interface-dominated toughening [96,100,107,122]	Excellent toughness–strength balance	Complex multiscale control is difficult
Porous/TPMS scaffolds	Biological porosity, permeability, low stress concentration [98,116]	Good mass transport and tissue integration	Fabrication complexity
Nanocellulose and peptide-based systems	Self-assembly, anisotropy, hierarchical order [103,123,124,125]	Sustainable, programmable, biologically compatible	Reproducibility and scale-up remain challenging

TPMS: Triply periodic minimal surfaces.

**Table 3:** Typical biomimetic surfaces, structures, and their applications.

Biomimetic Inspiration	Fabrication Method(s)	Structural Design	Application Area	Key Functional Outcome	Reference
Lotus leaf, Shark skin	Lithography, Etching, Deposition, Self-Assembly	Hierarchical micro/nano	Self-cleaning, Antifouling	Superhydrophobicity, Drag reduction	[128]
Collagen, Hydrogel	Soft Lithography	Micropatterned surfaces	Cell culture, Tissue engineering	Enhanced cell-biomaterial interaction	[149]
Moth-eye, Lotus leaf	Roll-to-roll Nanoimprint Lithography	Sub-200 nm arrays	Antireflection, Self-cleaning	Large-area, cost-effective production	[150]
Spiderweb, Bodhi leaf	Hierarchical Design, Additive Manufacturing	Multi-order honeycomb/tube	Energy absorption, Crashworthiness	SEA increases up to 282%	[133,134]
Bone, Dental tissue	Femtosecond Laser Texturing	Micro/nano surface roughness	Biomedical implants	Improved osseointegration, wettability	[151,152]
Lipid membranes, Virus-like particles	Microfluidic Continuous Flow	Controlled nano-assemblies	Drug delivery, Diagnostics	Uniform synthesis, scalable production	[153,154]

**Table 4:** Main applications and key limitations of nanofabrication in bionic devices.

Application Areas	What Nanofabrication Contributes	Key Limitations
Biosensors/bioelectronics	Higher surface area, better immobilization, improved sensitivity and specificity, flexible and wearable formats [111,112,118,121]	Scaling from lab prototypes to robust, reproducible devices remains difficult [121,143]
Drug delivery systems	Precise control of size, shape, reservoirs, and stimuli-responsiveness for controlled release [5,178]	Translational barriers include manufacturing cost, stability, and validation [5,182]
Tissue engineering/ biofabrication	Biomimetic microenvironments, patterned cell guidance, multiscale scaffolds, and integrated nanomaterials [114,115,126]	Precise nanomaterial integration and scale-up remain unresolved [114,126]
Organ-on-chip/lab-on-chip	Physiological flow control, microfluidic compartmentalization, and biomimetic tissue models [175,176,183]	Endpoint analysis and protocol inconsistency limit comparability [185]
Soft robotics/biohybrid systems	Multiscale actuation, flexible sensing, and biomimetic movement in compliant systems [186,187,190]	Sensing instability, degradation, and manufacturing limits still matter [112,186]

## Data Availability

The original contributions presented in this study are included in the article. Further inquiries can be directed to the corresponding author.
